# The role of double volume exchange transfusion in the management of severe hyperbilirubinemia while on extracorporeal life support in neonates: a case report

**DOI:** 10.3389/fped.2025.1570946

**Published:** 2025-05-21

**Authors:** Katherine Dalldorf, Courtney Juliano, Tenzin Dawa, Veniamin Ratner, Caterina Tiozzo

**Affiliations:** ^1^ Division of Neonatology, Department of Pediatrics, Icahn School of Medicine at Mount Sinai Hospital, New York, NY, United States; ^2^ CT Operating Room Clin Personnel, Icahn School of Medicine at Mount Sinai Hospital, New York, NY, United States

**Keywords:** ECMO - extracorporeal membrane oxygenation, newborn, hemolysis, severe hyperbilirubinemia, double volume exchange transfusion, case report

## Abstract

Extracorporeal membrane oxygenation (ECMO) related hemolysis is a severe complication seen frequently in neonates requiring ECMO support. While circuit exchange is a common management approach, it carries the risk of hemodynamic instability due to the inflammatory reaction triggered by exposure to a new circuit. We present the case of a newborn with remarkably elevated bilirubin levels that persisted despite circuit changes and responded to a double volume exchange transfusion (DVET) while under extracorporeal life support (ELS). This is the first reported occurrence of a DVET performed on a newborn with severe hemolysis while on veno-arterial ELS and only the second documented case in a pediatric patient. Our aim is to underscore the feasibility and safety of utilizing DVET for newborns undergoing ELS. This procedure serves as an alternative or adjunct approach to circuit replacement for the management of severe hemolysis and associated hyperbilirubinemia.

## Introduction

Hemolysis occurs in 10.5% of neonates requiring ELS (1) and, if severe, increases the risk of acute kidney injury, and is associated with longer hospital stays, and death (2, 3). The current approach to managing hemolysis is to replace the ECMO circuit in order to migitage ongoing hemolysis and/or lower bilirubin levels. However, this approach confers additional risk for infection and inflammation due to surface contact activation (4).

Double volume exchange transfusion (DVET) is an established therapeutic approach to management of severe hyperbilirubinemia in neonates (5). Therapeutic plasma exchange (TPE) has been reported in case series (6, 7) as an alternative to DVET for managing hemolysis during mechanical circulatory support. However, TPE requires specialized nursing and medical expertise and is typically available only in centers with such capabilities, whereas exchange transfusion is a simpler procedure with which most neonatologists are already familiar.

Despite the high incidence of ECMO-associated hemolysis in neonates, there is limited literature exploring the use of double-volume exchange transfusion while on ELS (8). To our knowledge, this is the first documented case of an infant under one month of age undergoing DVET for hemolysis while on ELS. This case report aims to showcase the success of this procedure and describe a potential approach for management of severe ECMO-related hemolysis. This manuscript was prepared following the CARE guidelines (https://www.care-statement.org).

## Case report

**Figure 1:**
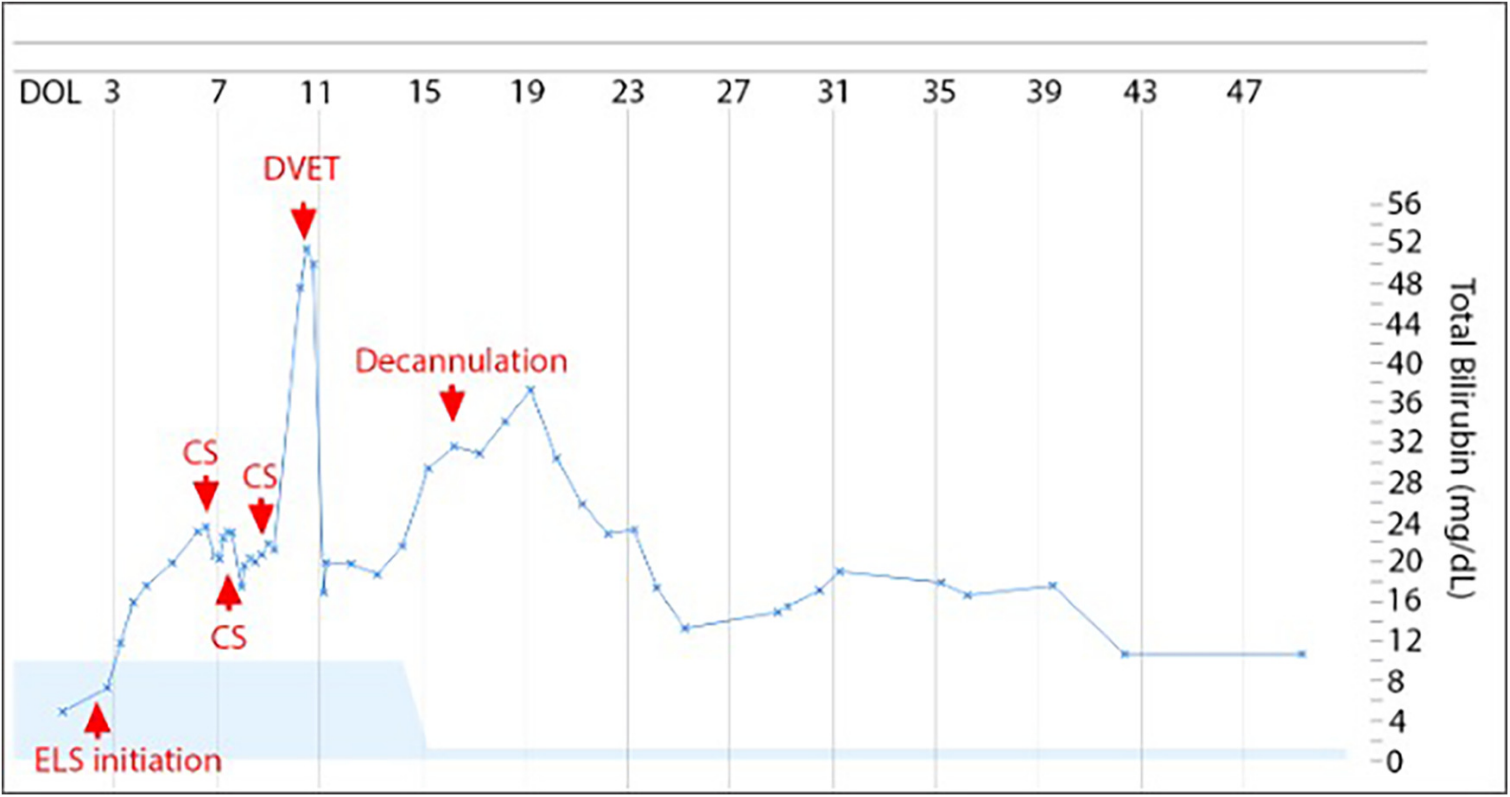
Total bilirubin levels trend. On the *x* axis, time (days of life). On the *y* axis, levels of total bilirubin (mg/dl). ECMO was started on DOL 3, circuit switched (CS) on DOL 6, 10, and 11, exchange transfusion on DOL 11 and decannulation on DOL 16. Light blue shading is normal range for total bilirubin adjusted for days of life.

This patient was a 38-week 2/7 days male prenatally diagnosed with left-sided congenital diaphragmatic hernia (CDH) with herniation of the entire colon, most of the small intestine, the stomach, and spleen and a lung area to Head circumference Ratio (LHR) of 0.88, portending a poor prognosis. ELS was initiated on DOL 3 for hypoxic respiratory failure despite maximum medical support. Infant was peripherally cannulated to veno-arterial ECMO using 8 Fr and 12 Fr Biomedicus cannulas. The ECMO circuit consisted of ¼ inch diameter SMART coated PVC tubing pack with a CARDIOHELP System (Maquet Medical Systems USA, Wayne, NJ) and a Quadrox-ID Adult PMP Oxygenator (Maquet Medical Systems USA, Wayne, NJ). A shunt was also inserted and diverted 500 ml/h post-oxygenated blood toward the venous limb of oxygenator circuit in order to maintain minimally recommended blood flow via oxygenator. The infant received bivalirudin anticoagulation to maintain an activated partial thromboplastin time (aPTT) target of 75–110 s and remained within the therapeutic range throughout the entire ECMO course. CDH was surgically repaired while on ECMO on day of life (DOL) 8. His ECMO run was notable for severe hemolysis beginning 24 h after cannulation (with a total bilirubin level of 17.6 mg/dl, initially managed with double phototherapy), despite the absence of known risk factors for hemolysis. The infant was Coombs negative, had normal G6PD activity, and there was no visible thrombus formation within the circuit or oxygenator. It was not possible to measure plasma free hemoglobin due to lab limitations, so hemolysis was quantified by rising bilirubin and qualified by color of skin and urine. Three circuit changes were performed (on DOL 6, 10 and 11) due to rapidly increasing total and direct bilirubin (Figure 1) without visible thrombi. Following the third circuit change, the infant became hypotensive requiring inotropic support and volume expansion, likely due to the inflammatory response associated with circuit change. Thus, the decision was made to perform a DVET through the ECMO circuit on DOL 11 instead of completing a fourth circuit change.

**Figure 2:**
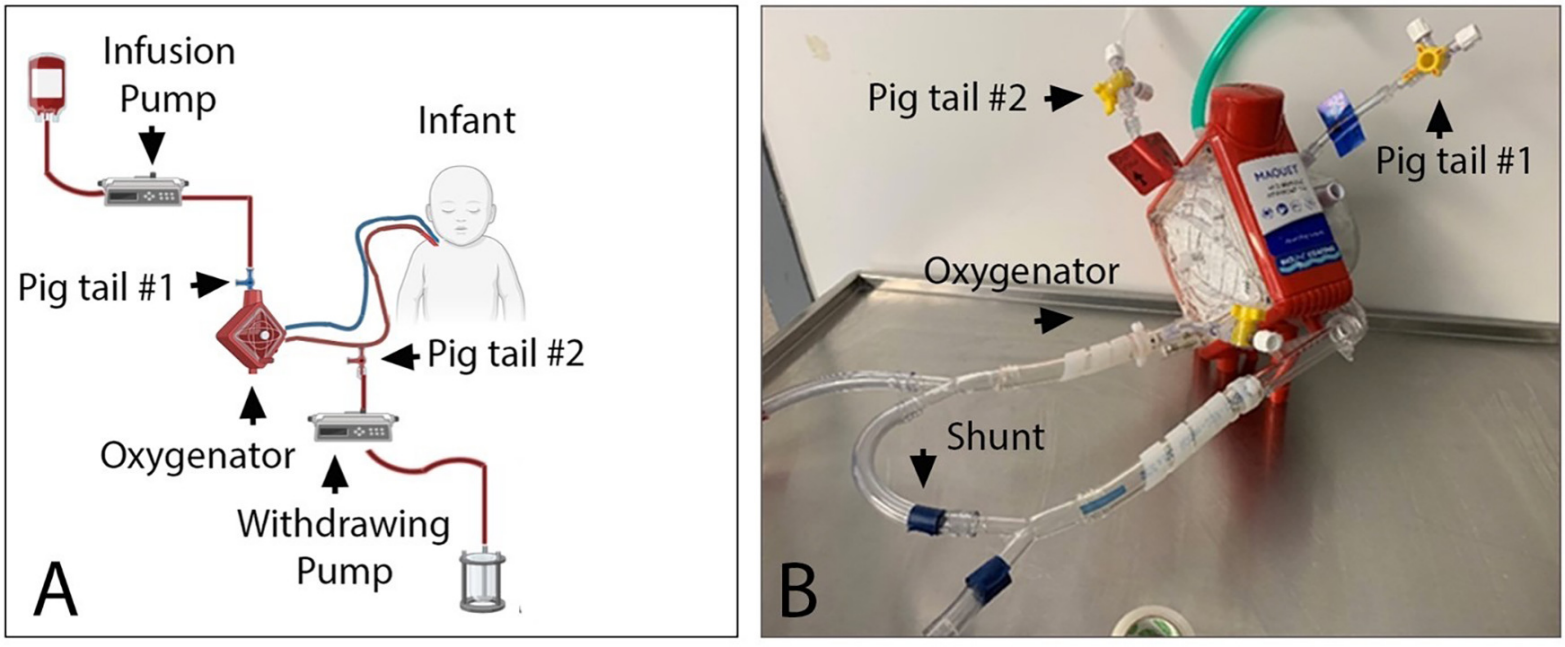
Schema **(A)** and picture **(B)** of the DVET set up during neonatal ECMO run. Blood was infused into the ECMO circuit using infusion pump connected to the venous side of the oxygenator via a pig tail #1. Blood was withdrawn from the circuit using withdrawing pump connected to the arterial side of the oxygenator via pig tail #2. Euvolemia was maintained by setting both pumps at the same rate. A shunt was used in order to maintain minimally recommended blood flow of 500ml/min via Cardiohelp oxygenator.

The exchange transfusion volume was determined based on the patient's blood volume (80 ml/kg) plus the volume of the ECMO circuit (500 ml) and calculated to be 1,500 ml of irradiated, CMV-negative packed red blood cells reconstituted in plasma for a final hematocrit of 45%. Two pigtails were placed: one just before the oxygenator to infuse blood, and the other post-oxygenator to withdraw blood (Figure 2). DVET was performed isovolumetrically by infusing and withdrawing blood at the same rate over 4 h. This was started at a lower rate of 300 ml/h for one hour to ensure the patient would tolerate the procedure, and then increased to 400 ml/h for the remaining 3 h. Vital signs were continuously monitored without any hemodynamic changes. There was no change noted in trans-oxygenator pressure or mean arterial pressure, and ECMO flow remained constant throughout the procedure. Fresh frozen plasma (10 ml/kg x2) and platelet (10 ml/kg x2) transfusions were administered based on predetermined platelet and coagulation profile targets. Arterial blood gases were done every 30 min, and a chemistry, bilirubin, and complete blood count were sampled at the initation, midpoint, and conclusion of the DVET.

Immediately before DVET, total bilirubin was 50.0 mg/dl and unconjugated bilirubin was 24.4 mg/dl. One hour after the procedure, total bilirubin had decreased to 16.9 mg/dl and unconjugated bilirubin to 7.7 mg/dl. The infant remained on ELS support for 6 days following the procedure and then was successfully decannulated, at which point the total bilirubin level had risen to 31.6 mg/dl but remained below the pre-exchange level. The infant was discharged home on DOL 81 on room air and partial gavage feeds. Total and direct bilirubin were 3.1 mg/dl and 2.2 mg/dl, respectively, prior to discharge. Head ultrasound on DOL 35 was normal and no additional head imaging was done. Follow up examination at two years of age demonstrated that the infant was developing appropriately for age with a mild expressive language delay, for which he was receiving Early Intervention therapy. At this time, a Bayley Scales of Infant and Toddler Development (Fourth Edition) was administered, during which he scored in the 63rd percentile on Cognitive Scale, 23rd percentile on Language Scale, and in the 34th percentile on Motor Scale.

## Discussion

This case supports the use of DVET for ECMO-induced severe hyperbilirubinemia during the neonatal period, and suggests that it is a safe and feasible alternative to circuit exchange. In this case of refractory hyperbilirubinemia, DVET proved more effective than circuit change in lowering the bilirubin level with the additional benefit of reducing morbidity associated with ciruit change induced systemic inflammatory response. This is the first case report outlining use of DVET for treatment of ECMO-induced hemolysis/hyperbilirubinemia in a neonate; previously, the youngest reported case to received DVET during an ECMO run was a 54 days old infant (8).

In this case, though there was not detectable thrombus burden on the circuit components, the circuit was exchanged 3 times in an attempt to mitigate hemolysis and lower bilirubin levels before DVET was considered on ELS. The decision to ultimately attempt exchange transfusion was driven by 2 factors: (1) earlier circuit change on the same day had not effectively slowed the rising bilirubin levels and (2) earlier circuit change on the same day had not been well-tolerated and was associated with significant hemodynamic changes, namely hypotension and decreased urine output.

This case affirms that DVET in a patient on ELS can be well-tolerated and effective for use in treating hyperbilirubinemia. The prior reported case (8) outlined use of DVET in an older infant (54 days old) on ELS, and in our configuration, two pigtails were utilized as opposed to a manifold, highlighting the safety and efficacy profile across a variety of clinical circumstances and identifying alternative approaches to circuit configuration.

While DVET on ELS may be a valuable treatment approach to severe hyperbilirubinemia, reported experience remains limited. Further study is required to characterize additional potential risks and benefits and to identify those clinical scenarios in which DVET may be indicated over circuit exchange for management of hyperbilirubrubinemia on ELS.

## Conclusion

This case affirms that the use of DVET for ECMO-induced severe hyperbilirubinemia during the neonatal period is a safe and feasible alternative to circuit exchange.

## Data Availability

The original contributions presented in the study are included in the article/Supplementary Material, further inquiries can be directed to the corresponding author.
